# Relevance of the International Study Group of Pancreatic Surgery and the Dutch Pancreatic Cancer Group Classifications of Pancreas-Specific Risk Factors in Predicting Clinically Relevant Postoperative Pancreatic Fistula in the Whipple Procedure

**DOI:** 10.7759/cureus.84051

**Published:** 2025-05-13

**Authors:** Saurav Karki, Bishnu Kandel, Deepak Sharma, Nishnata Koirala, Paleswan Joshi Lakhey

**Affiliations:** 1 Department of Surgical Gastroenterology, Tribhuvan University Teaching Hospital, Maharajgunj Medical Campus, Institute of Medicine, Kathmandu, NPL

**Keywords:** clinically-relevant postoperative pancreatic fistula, dutch 3 tier classification, isgps classification, main pancreatic duct diameter, pancreas specific risk factors, pancreaticoduodenectomy, post operative pancreatic fistula, soft pancreas

## Abstract

Background

The International Study Group of Pancreatic Surgery (ISGPS)introduced a four-tier classification system, including pancreatic texture and pancreatic duct diameter, to aid the risk stratification of clinically relevant postoperative pancreatic fistula. The Dutch Pancreatic Cancer Group (DPCG) validated the ISGPS risk classification and proposed a three-tier classification system. This study was conducted to compare the clinically relevant postoperative pancreatic fistula rate among two classification systems.

Methods

This study was conducted by a retrospective review of the prospectively maintained data of 165 patients who underwent pancreaticoduodenectomy, also known as the Whipple Procedure, between 2015 and 2024 in a single unit of the Department of Surgical Gastroenterology at Tribhuvan University Teaching Hospital, Kathmandu, Nepal. The preoperative, intraoperative, and postoperative variables were analyzed to assess the relevance of the two classifications to predict clinically relevant postoperative pancreatic fistula.

Results

Ampullary carcinoma was the most common indication of pancreaticoduodenectomy (47.3%, n=78). Fifty patients (30.3%) had a main pancreatic duct diameter ≤3 mm, and 62.4% (n=103) had soft pancreatic texture. Twenty-eight patients (17.0%) developed clinically relevant postoperative pancreatic fistula, 44 (26.7%) had major complications (Clavien Dindo ≥3), and in-hospital mortality was seen in 13 (7.9%). Main pancreatic duct diameter ≤3 mm (36.0% vs 8.7%, P: <0.001), blood loss ≥500 ml (21.4% vs 7.5%, P: 0.027), and non-pancreatic pathology (21.5% vs 4.5%, P: 0.010) were significantly associated with clinically relevant postoperative pancreatic fistula but main pancreatic duct diameter ≤3 mm (OR: 7.313, P: 0.007, 95%CI: 1.462-12.124) was the only independent predictor. The rate of clinically relevant postoperative pancreatic fistula was significantly different in the subclasses in both the ISGPS and the DPCG classifications, being highest in Type D (40.5%, n=17). Both the classification systems showed similar predictivity for clinically relevant postoperative pancreatic fistula, with similar area under the curve, 0.707 for the ISGPS classification and 0.710 for the DPCG classification.

Conclusion

This study showed that the Type D (as per the ISGPS classification) or the two-risk-factor group (as per the DPCG classification) has the highest rate of postoperative complications after pancreaticoduodenectomy. On further analysis of the classification of pancreas-specific risk factors, including pancreatic texture and main pancreatic duct diameter, according to the ISGPS and DPCG classification systems, predictive accuracy was similar for clinically relevant postoperative pancreatic fistula; however, the DPCG classification with the simpler three-tier system is easier to apply in practice.

## Introduction

Pancreaticoduodenectomy (PD), also known as the Whipple Procedure, remains one of the most complex abdominal surgeries done for malignant disease of the head of the pancreas, periampullary region, and for selected benign diseases. Postoperative outcome after PD has improved in terms of mortality, which has decreased to less than 5%, but morbidity remains high (up to 50%) [1,2]. Clinically relevant postoperative pancreatic fistula (CR-POPF) is the major determinant of postoperative mortality and morbidity [3-5]. Multiple risk factors, including patient-related, disease-related, or pancreas-related, have been deemed relevant in the development of CR-POPF [6,7].

Multiple risk scoring systems for predicting the development of CR-POPF have been developed and validated, like the original Fistula Risk Score (oFRS), alternate Fistula Risk Score (aFRS), and updated alternate Fistula Risk Score (uaFRS) [8-11]. Among the risk factors, pancreas-specific risk factors, which include the pancreatic texture and main pancreatic duct diameter (MPD) at the transection site, are found to be consistently associated with the development of CR-POPF and thus are included in most of the risk scoring models.

The International Study Group of Pancreatic Surgery (ISGPS) classified these pancreas-specific risk factors (pancreatic texture and MPD) and proposed a four-tier system of classification as Type A (Texture: not-soft and duct diameter: >3 mm), Type B (Texture: not-soft and duct diameter: ≤3 mm), Type C (Texture: soft and pancreatic duct: >3 mm), and Type D (Texture: soft and pancreatic duct: ≤3 mm) to risk stratify patients undergoing PD for development of CR-POPF [6]. The Dutch Pancreatic Cancer Group (DPCG) reviewed their nationwide Dutch Pancreatic Cancer Audit and validated the ISGPS classification system, showing increasing risk of development of CR-POPF from Type A to D. Meanwhile, they found that group B and group C did not have any significant difference in risk for development of CR-POPF and thus proposed a simpler three-tier system of classification [12]. 

This study was conducted with the aim of assessing the relevance of the ISGPS classification in our population and to compare it with the simpler DPCG three-tier classification system.

The preliminary result of this study with 130 patients was presented as an oral paper at the Asian-Pacific Hepato-Pancreato-Biliary Association (A-PHPBA) conference in Bangalore, India, on September 28, 2023.

## Materials and methods

This was an observational cross-sectional study done by the retrospective review of a prospectively maintained database. It was conducted at the Department of Surgical Gastroenterology, Tribhuvan University Teaching Hospital, Maharajgunj Medical Campus, Institute of Medicine, Kathmandu, Nepal. The study was approved by the Institutional Review Committee of Tribhuvan University, Institute of Medicine (approval number: 449(6-11)E2).

Study cohort

All patients who underwent open PD between 2015 and 2024 in a single unit of the Department of Surgical Gastroenterology at Tribhuvan University Teaching Hospital were included. Patients who died before postoperative day 3 were excluded, as they could not be classified for POPF as per the ISGPS classification. A total of 165 patients were thus included in the study.

Data collection

Demographic data in terms of age, sex, comorbidities, BMI, American Society of Anesthesiologists (ASA) physical status, and preoperative blood investigation were collected. Likewise, intraoperative variables like site of lesion, pancreatic texture, MPD, duration of surgery, blood loss, techniques of pancreaticoenteric anastomosis, and use of octreotide were noted. The technique of pancreatoenteric anastomosis was dunking pancreaticojejunostomy for the first five years and later switched to modified Blumgart pancreaticojejunostomy using polypropylene 3-0 for trans-pancreatic stitch and polydioxanone 5-0 for duct to mucosa anastomosis. 

Definitions and measurements

Pancreatic texture was defined as either soft or non-soft by palpation by the operating surgeon, and MPD was measured at the remnant pancreatic end at the transection site. Postoperative pancreatic fistula (POPF) was defined and graded as per the ISGPS 2016 classification, and Grade B and C POPF were grouped as CR-POPF [13]. Likewise, postpancreatectomy hemorrhage (PPH), delayed gastric emptying (DGE), chyle leak, and bile leak were noted, defined, and graded as per the respective ISGPS and International Study Group of Liver Surgery (ISGLS) classifications [14-17]. Other postoperative complications were graded as per Clavien-Dindo classification for individual complications [18,19]. Major complications were classified as Clavien-Dindo grade ≥3. 

To compare the two classification systems of ISGPS and DPCG, patients were categorized into four groups as per the ISGPS classification and reclassified by combining types B and C to form the three risk categories as per the DPCG classification. Accordingly, patients with no risk factors were categorized as group A, those with either of the two risk factors as group B, and patients with both risk factors as group C.

Data analysis

Baseline characteristics comprising continuous data were analyzed and expressed in terms of mean with standard deviations and median with interquartile range (IQR) and tested using the independent t-test or Mann-Whitney U test. The categorical data were expressed in frequencies and percentages and analyzed with the Fisher exact test or chi-square test, whichever was appropriate. Rates of pancreas-specific complications, including CR-POPF, PPH, DGE, other postoperative complications (bile leak, surgical site infection (SSI)), Clavien Dindo grade, and outcomes in terms of in-hospital mortality and hospital stay were calculated for the ISGPS four-tier risk categories and also for the DPSG three-tier system. Both classification systems were compared for CR-POPF using the receiver operating characteristic (ROC) curve, and the area under the curve (AUC) was calculated, and sensitivity and specificity were calculated for both classification systems. Statistical analysis was done using IBM SPSS Statistics for Windows, Version 25.0 (Released 2017; IBM Corp., Armonk, New York, United States).

## Results

During the study period of 10 years, a total of 165 patients underwent PD out of which 105 (63%) were male, and the mean age was 55.16±13.18 years. A total of 58 patients (35.2%) underwent preoperative biliary drainage. Ampullary carcinoma was the most common indication of PD, accounting for 78 cases (47.3%), followed by carcinoma head of the pancreas, distal cholangiocarcinoma, and duodenal carcinoma. Of the patients, 103 (62.4%) had soft pancreas, and 50 (30.3%) had MPD ≤3 mm (Table 1). Out of 165 patients, 28 (17.0%) developed CR-POPF, of which 12 (7.3%) were grade B and 16 (9.7%) were grade C (CR-POPF). Likewise, 22 (13.3%) developed PPH, 12 (7.3%) had DGE, and two patients had bile leak. A total of 129 patients (78.2%) had some sort of complications during the postoperative period, of which 44 (26.7%) had major complications as per the Clavien-Dindo classification, and in-hospital mortality occurred in 13 patients (7.9%).

**Table 1:** Preoperative, intraoperative, and postoperative variables in the study cohort (N=165) BMI: body mass index; DTM: duct to mucosa; CR-POPF: clinically relevant postoperative pancreatic fistula; PPH: post pancreatectomy hemorrhage; DGE: delayed gastric emptying; CD: Clavien-Dindo

Variables	Values
Age (years), mean±SD	55.41 ± 13.05
Gender, male, n (%)	105 (63.6%)
Diagnosis, n (%)	
Ampullary carcinoma	78 (47.3%)
Carcinoma head of pancreas	44 (26.7%)
Distal cholangiocarcinoma	25 (15.2%)
Duodenal carcinoma	11(6.7%)
Others	7 (4.2%)
Body mass index (BMI), mean±SD	21.21 ± 3.82
Comorbidities (yes), n (%)	63 (38.2%)
Preoperative total bilirubin, mean± SD, (µmol/L)	149 ± 157.83
Preoperative serum albumin, mean±SD, (mg/dl)	35.62 ±7.5
Preoperative biliary drainage (yes), n (%)	58 (35.2%)
Duration of surgery (minutes), mean±SD	374.73 ± 67.56
Intraoperative blood loss (ml), mean±SD	536.15 ± 241.44
Pancreatic texture, n (%)	
Soft	103 (62.4%)
Non-soft	62 (37.6%)
Main pancreatic duct diameter, n (%)	
≤ 3mm	50 (30.3%)
> 3mm	115 (69.7%)
Type of pancreaticoenteric anastomosis, n (%)	
Modified Blumgart DTM	86 (52.1%)
Dunking	79 (47.9%)
Original fistula risk score	
Negligible	9 (5.5%)
Low	34 (20.6%)
Intermediate	110 (66.7%)
High	12(7.3%)
CR-POPF, n (%)	28(17.0%)
B	12(7.3%)
C	16 (9.7%)
PPH, n (%)	22 (13.3%)
DGE, n (%)	12 (7.3%)
Bile leak, n (%)	2 (1.5%)
Surgical site infection, n (%)	64 (38.8%)
Major complication, CD ≥ 3, n (%)	44 (26.7%)
In hospital mortality, n (%)	13 (7.9%)
Length of hospital stay in days, mean±SD	14.72 ± 9.04

When the 165 patients were categorized as per the ISGPS four-tier classification, 54 (32.7%) were in Type A, eight (4.8%) in Type B, 61 (37%) in Type C, and 42 (25.5%) in Type D (Table 2). Likewise, on categorization as per the DPSG classification, the number of patients in the first and last types remained the same, while types B and C were combined to constitute 69 patients (41.8%) (Table 3). The major demographics and preoperative variables, like age, gender, and preoperative biliary drainage, were found to be symmetrically distributed among the different groups in both classification systems. However, the primary pathology requiring PD and oFRS had a significantly different distribution in both classifications.

**Table 2:** Preoperative variables in the four-tier ISGPS classification (N=165) FRS: Fistula Risk Score; ISGPS: International Study Group of Pancreatic Surgery

Variables	Category	Total number of patients	Type A (non-soft, >3 mm) (n=54), n (%)	Type B (non-soft, ≤3 mm) (n=8), n (%)	Type C (soft, >3 mm) (n=61), n (%)	Type D (soft, ≤3 mm) (n=42), n (%)	Chi-square value	P value
Age	< 60	96	31 (57.4%)	4 (50%)	31 (50.8%)	30 (71.4%)	6.458	0.191
	≥ 60	69	23 (42.6%)	4 (50%)	30 (49.2%)	12 (28.6%)		
Gender	Male	105	37 (68.5%)	7 (87.5%)	39 (63.9%)	22 (52.4%)	4.483	0.164
	Female	60	17 (31.5%)	1 (12.5%)	22 (36.1%)	20 (47.6%)		
Pathology	Non-pancreatic	121	28 (51.9%)	5 (62.5%)	52 (85.2%)	36 (85.7%)	21.874	< 0.001
	Pancreatic	44	26 (48.1%)	3 (37.5%)	9 (14.8%)	6 (14.3%)		
Preop biliary drainage	Yes	58	20 (37.1%)	4 (50%)	19 (31.1%)	15 (35.7%)	2.371	0.737
	No	107	34 (62.9%)	4 (50%)	42 (68.9%)	27 (64.3%)		
Original FRS	< 7	153	54 (100%)	8 (100%)	59 (96.7%)	32 (76.2%)	23.337	<0.001
	≥ 7	12	0 (0%)	0 (0%)	2 (3.3%)	10 (23.8%)		

**Table 3:** Preoperative variables in the three-tier DPCG classification (N=165) FRS: Fistula Risk Score; DPCG: Dutch Pancreatic Cancer Group

Variables	Category	Total number of patients	Group A (no risk factor) (n=54), n (%)	Group B (one risk factor) (n=69), n (%)	Group C (two risk factors) (n=42), n (%)	Chi-square value	P value
Age	< 60	96	31 (57.4%)	35 (50.7%)	30 (71.4%)	5.506	0.099
	≥ 60	69	23 (42.6%)	34 (49.3%)	12 (28.6%)		
Gender	Male	105	37(68.5%)	46 (66.7%)	22 (52.4%)	3.196	0.209
	Female	60	17 (31.5%)	23 (33.3%)	20 (47.6%)		
Pathology	Non-pancreatic	121	28 (51.9%)	57 (82.6%)	36 (85.7%)	21.261	< 0.001
	Pancreatic	44	26 (48.1%)	12 (17.4%)	6 (14.3%)		
Preop biliary drainage	Yes	58	20 (37.1%)	23 (33.3%)	15 (35.7%)	0.250	0.909
	No	107	34 (62.9%)	46 (66.7%)	27 (64.3%)		
Original FRS	< 7	153	54 (100%)	67 (97.1%)	32 (76.2%)	23.237	< 0.001
	≥ 7	12	0 (0%)	2 (2.9%)	10 (23.8%)		

The incidence of CR-POPF was five (9.3%) patients in Type A, while Type D had the highest incidence, including 17 (40.5%) patients, with a statistically significant difference among the groups (Table 4). The incidence of PPH, DGE, SSI, and reoperation showed similar distribution across the groups. However, Type D had the highest rate of major complications with 19 (45.2%) patients, followed by Type B, Type A, and Type C, accounting for two (25%), 13 (24.1%), and 10 (16.4%) patients, respectively. The in-hospital mortality rate was highest in Type D, with seven (16.7%) patients, while none of the patients in Type C died.

**Table 4:** Postoperative outcomes in the four-tier ISGPS classification (N=165) ISGPS: International Study Group of Pancreatic Surgery; CR-POPF: clinically relevant postoperative pancreatic fistula; PPH: postpancreatectomy hemorrhage; DGE: delayed gastric emptying; SSI: surgical site infection; CD: Clavien-Dindo

Variables	Category	Total number of patients	Type A (non-soft, >3 mm) (n=54), n (%)	Type B (non-soft, ≤3 mm) (n=8), n (%)	Type C (soft, >3 mm) (n=61), n (%)	Type D (soft, ≤3 mm) (n=42), n (%)	Chi-square value	P value
CR-POPF	Yes	28	5 (9.3%)	1 (12.5%)	5 (8.2%)	17 (40.5%)	22.262	<0.001
	No	137	49 (90.7%)	7 (87.5%)	56 (91.8%)	25 (59.5%)		
PPH	Yes	22	6 (11.1%)	1 (12.5%)	5 (8.2%)	10 (23.8%)	5.667	0.132
	No	143	48 (88.9%)	7 (87.5%)	56 (91.8%)	32 (76.2%)		
DGE	Yes	12	5 (9.3%)	0 (0%)	2 (3.3%)	5 (11.9%)	3.598	0.293
	No	153	49 (90.7%)	8 (100%)	59 (96.7%)	37 (88.1%)		
Reoperation	Yes	13	4 (7.4%)	0 (0%)	2 (3.3%)	7 (16.5%)	6.874	0.070
	No	152	50 (92.6%)	8 (100%)	59 (96.7%)	35 (83.3%)		
SSI	Yes	64	20 (37.0%)	2 (25%)	22(36.1%)	20 (47.6%)	3.156	0.216
	No	101	34 (63.0%)	6 (75%)	39 (63.9%)	22 (52.4%)		
CD ≥3	Yes	44	13 (24.1%)	2 (25%)	10 (16.4%)	19 (45.2%)	10.971	0.012
	No	121	41 (75.9%)	6 (75%)	51 (83.6%)	23 (54.8%)		
Outcome	Death	13	5 (9.3%)	1 (12.5%%)	0 (0%)	7 (16.7%)	10.193	0.018
	Discharged	152	49 (90.7%)	7 (87.5%)	61 (100%)	35 (83.3%)		

The categorization of patients as per the three-tier classification system proposed by DPCG had a similar association in terms of postoperative complications (Table 5). Like in the ISGPS classification, the three-tier DPCG classification system also revealed the difference in the occurrence of CR-POPF among the three groups, reaching the level of significance (P<0.001). In both classification systems, statistically significant differences were observed for in-hospital mortality and major complications, but in the DPCG classification, an additional significant difference was found for reoperation rate.

**Table 5:** Postoperative outcomes in the three-tier DPCG classification (N=165) ISGPS: International Study Group of Pancreatic Surgery; CR-POPF: clinically relevant postoperative pancreatic fistula; PPH: postpancreatectomy hemorrhage; DGE: delayed gastric emptying; SSI: surgical site infection; CD: Clavien-Dindo

Variables	Category	Total number of patients	Group A (no risk factor) (n=54), n (%)	Group B (one risk factor) (n=69), n (%)	Group C (two risk factors) (n=42), n (%)	Chi-square value	P value
CR-POPF	Yes	28	5 (9.3%)	6 (8.7%)	17 (40.5%%)	22.096	<0.001
	No	137	49 (90.7%)	63 (91.3%)	25 (59.5%)		
PPH	Yes	22	6 (11.1%)	6 (8.7 %)	10 (23.8%)	5.466	0.064
	No	143	48 (88.9%)	63 (91.3%)	32 (76.2%)		
DGE	Yes	12	5 (9.3%)	2 (2.9%)	5 (11.9%)	3.498	0.164
	No	153	49 (90.7%)	67 (97.1%)	37 (88.1%)		
Reoperation	Yes	13	4 (7.4%)	2 (2.9%)	7 (16.7%)	6.781	0.033
	No	152	50 (92.6%)	67 (97.1%)	35 (83.3%)		
SSI	Yes	64	20 (37.0%)	24 (34.8%)	21(47.6%)	3.066	0.384
	No	101	34 (63.0%)	45 (65.2%)	22 (52.4%)		
CD ≥3	Yes	44	13 (24.1%)	12 (17.4%)	19 (45.2%)	10.495	0.005
	No	121	41 (75.9%)	57 (82.6%)	23 (54.8%)		
Outcome	Death	13	5 (9.3%)	1 (1.4%)	7 (16.7%)	8.428	0.014
	Discharged	152	49 (90.7%)	68 (98.6%)	35 (83.3%)		

**Figure 1:**
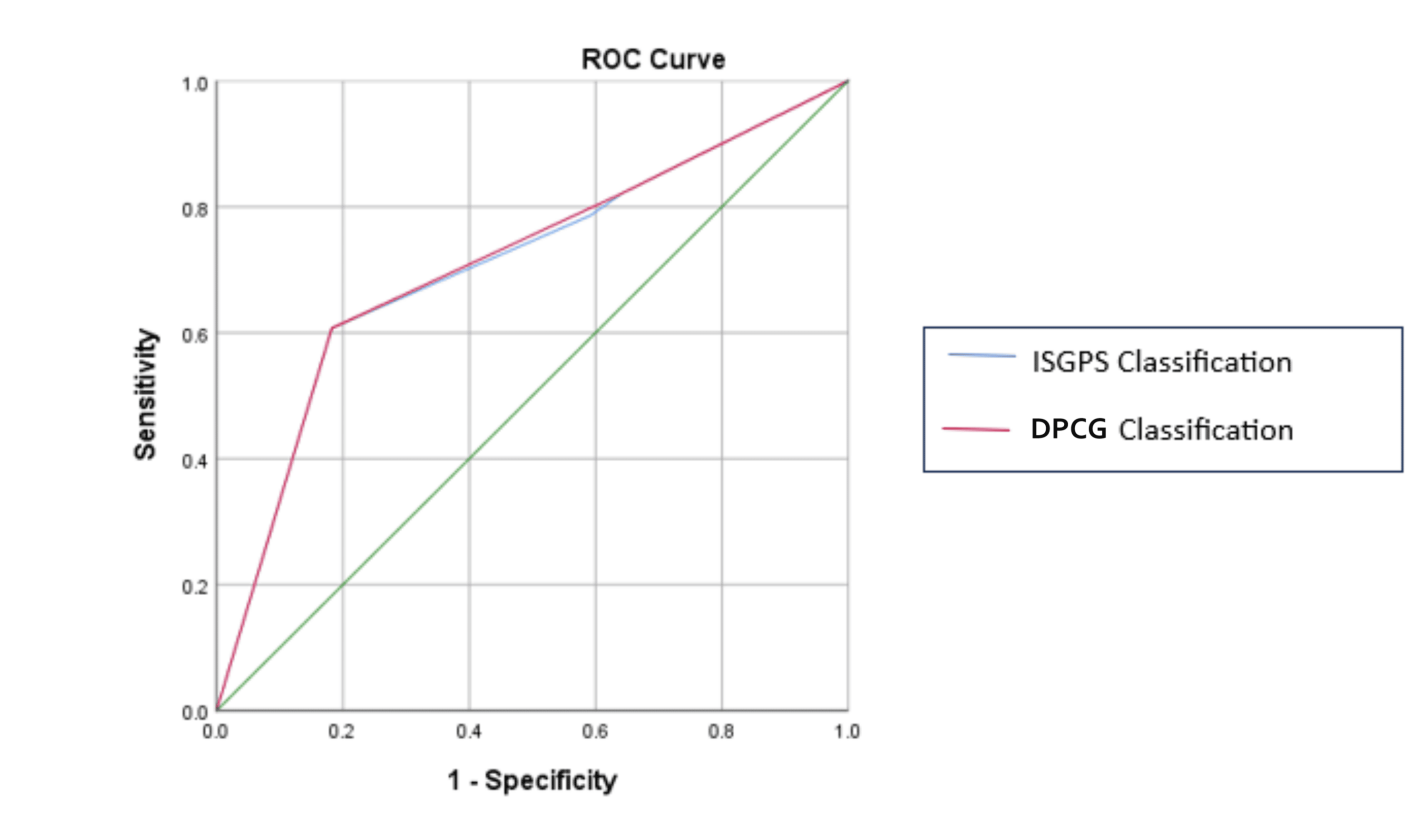
ROC curve ROC: receiver operating characteristics; ISGPS: International Study Group for Pancreatic Surgery; DPCG: Dutch Pancreatic Cancer Group

The efficacy of both the classification systems in predicting CR-POPF was comparable, with AUC being 0.707 for the ISGPS four-tier classification system and 0.710 for the DPCG three-tier classification system. Figure 1 shows the ROC curve, and Table 6 compares the predictive accuracy of ISGPS and DPCG classification systems.

**Table 6:** Comparison of predictive accuracy of ISGPS and DPCG classification system ISGPS: International Study Group for Pancreatic Surgery; DPCG: Dutch Pancreatic Cancer Group

Classification system	Area	Std Error	Asymptomatic Sig	95% Confidence Interval	
				Lower bound	Upper Bound
ISGPS four-tier category	0.707	0.059	0.001	0.591	0.822
DPCG three-tier category	0.710	0.059	0.000	0.595	0.825

On bivariate analysis of the preoperative and intraoperative risk factors for the development of CR-POPF, non-pancreatic pathology, blood loss >500 ml, and MPD ≤3 mm were significantly associated, while soft texture of pancreas (p value: 0.053) and dunking pancreaticojejunostomy (p value: 0.056) had a borderline level of significance (Table 7). oFRS was also significantly associated with the development of CR-POPF. In multivariate analysis, only MPD ≤3 mm showed a significant association.

**Table 7:** Bivariate and multivariable analysis of risk factors for CR-POPF (N=165) CR-POPF: clinically relevant postoperative pancreatic fistula; BMI: body mass index; MPD: main pancreatic duct; oFRS: original fistula risk score Statistical analysis with Chi-square test and multivariate analysis for variables with P<0.1

Variable	Total number of patients	CR-POPF (n=28), n (%)	No CR-POPF (n=137), n (%)	Chi square test	P value	Multivariate analysis		
						OR	95% CI	p-value
Age (≥60yrs)	69	12 (68.9%)	57 (41.6%)	0.015	0.903	-	-	-
Sex (Male)	105	18 (64.3%)	87 (63.5%)	0.006	0.938	-	-	-
BMI (≥25)	21	3 (10.7%)	18 (13.1%)	0.021	0.965	-	-	-
Preop Diagnosis (Non-pancreatic)	121	26 (92.8%)	95 (69.3%)	6.573	0.010	2.024	0.641 – 16.403	0.155
Preop Drainage (Yes)	58	11 (39.3%)	47 (34.3%)	6.251	0.615	-	-	-
Co-morbidity (Yes)	63	10 (35.7%)	53 (38.7%)	0.087	0.768	-	-	-
Blood Loss (≥500 ml)	112	24 (85.7%)	88 (64.2%)	4.920	0.027	3.552	0.090 – 1.048	0.059
Operating Time (≤390 minutes)	85	15 (53.6%)	70 (51.1%)	0.057	0.811	-	-	-
Pancreatic Texture (Soft)	103	22 (78.6%)	81 (59.1%)	3.748	0.053	1.117	0.334 – 3.230	0.948
MPD ≤ 3mm	50	18 (64.3%)	32 (23.4%)	18.439	<0.001	7.313	1.462 – 12.124	0.007
oFRS ≥ 7	12	7 (25%)	5 (3.6%)	15.714	<0.001	2.073	0.081 – 1.865	0.150
Anastomosis (Dunking)	79	18 (64.3%)	61 (44.5%)	3.638	0.056	1.309	0.647-5.247	0.253
Octreotide (Yes)	83	18 (64.3%)	65 (47.5%)	2.637	0.104	-	-	-

## Discussion

Pancreas-specific risk factors are among the major predictors of POPF following PD. Along with pancreatic texture and pancreatic duct diameter, pancreatic pathology, pancreatic thickness at the transection site, and acinar cell content are other pancreas-related factors found to have an association with POPF [20-23]. To objectify the risk with pancreas-specific risk factors, Anorsge et al. categorized patients according to pancreatic duct diameter and pancreatic texture and established their association with POPF [24], which was further replicated by ISGPS and DPCG studies on the classification of pancreatic risk factors [6,12]. The current study with 165 patients with PD reproduced the higher CR-POPF rate in Type D as compared to other groups, suggesting that pancreas-specific risk factors represented by pancreatic duct diameter and pancreatic texture are major determinants of CR-POPF. However, the gradual increase in CR-POPF rate from Type A to Type D, as seen in the ISGPS and DPCG studies [6,12], was not replicated in this study.

Studies by Anorsge et al., ISGPS, and DPCG established the nonuniform distribution of patients in different groups [24,6,12]. The current study not only replicated but further emphasized the non-uniform distribution with the least number of patients (4.8%, n=8) in the ISGPS Type B group (non-soft, ≤3 mm). Thus, combining Group B and Group C, each with at least one risk factor, appeared to be more logical and also produced statistically significant results not only for CR-POPF but also for reoperation rate, major complications (Clavien Dindo Grade≥3), and mortality. However, in terms of CR-POPF, the performance of the three-tier Dutch classification (AUC 0.710, SE .059) did not have a statistically significant difference as compared to the ISGPS four-tier classification (AUC 0.707, SE .059), suggesting that the three-tier classification, being simpler, would be a justifiable alternative; however, this has to be validated with more robust prospective studies.

Ampullary carcinoma constituted almost half of the total patients (47.3%, n=78) while carcinoma head of the pancreas constituted 26.7% (n=44) of the patients. Although the most common indication of PD is carcinoma head of the pancreas, there is some regional difference, with some studies, particularly from Asian countries, revealing a higher incidence of periampullary lesions [2,25]. Karim et al., in their study of outcomes after PD, reported ampullary pathology accounting for 43.88% while carcinoma head of the pancreas accounted for 16.33% [26].

In contrast to the ISGPS classification study [6], the current study had a higher number of patients in type C (37% vs 17.7%). Since the number of patients with ampullary pathology was high, and as ampullary lesions are more likely to have soft pancreas, this could be the logical explanation for more patients being in Type C. Yang et al. in their study also showed that the patients with ampullary pathology had soft pancreas and smaller main pancreatic duct diameter as compared to those with non-ampullary pathology [27]. Likewise, Kuesters et al. in their study revealed a statistically significant higher incidence rate of CR-POPF (30.7% vs. 16.8%, p value < 0.001) in patients undergoing PD for ampullary carcinoma as compared to carcinoma head of the pancreas [28]. This could be one of the reasons for the higher overall rate of CR-POPF (17.0%, n=28) and mortality (7.9%, n=13) in the current study as compared to recent data. 

Except for pancreatic texture, the other predictors of CR-POPF as per oFRS [9], like pathology, intraoperative blood loss, and pancreatic duct diameter, are significantly associated with CR-POPF in the current study also. In contrast to most of the studies [6,10,12,29], pancreatic duct size rather than texture had a higher OR and level of significance for CR-POPF, and MPD ≤3 mm remained as the only significant preoperative variable in multivariate analysis. Assessment of texture is subjective and has individual variations, which might have added to the different results.

One of the striking findings in the current study was that although the rate of CR-POPF and PPH was similar between Type A and C as per the ISGPS classification, DGE, major complications, reoperation rate, and mortality were lower in Type C than Type A in the ISGPS classification. Pancreatic pathology constituted a major bulk of the Type A subgroup (48.1%, n=26) as compared to the Type C subgroup (14.8%, n=9). Pancreaticoduodenectomy for pancreatic pathology usually requires more extensive dissection and extended lymphadenectomy as compared to that for other periampullary cancers. This leads to increased intraoperative time and greater blood loss. These factors contribute to a higher likelihood of postoperative complications. As Type A had a higher number of patients with pancreatic pathology, this could be the reason for the higher number of complications in this group as compared to Type C, where most of the patients had non-pancreatic pathology.

Limitations of the study

Some limitations of this study are its retrospective nature, which naturally comes with inherited selection bias. Additionally, the treatment protocol evolved over time, particularly in terms of pancreatojejunostomy anastomosis. In the initial phase, the Dunking technique was used, but since 2021, the Modified Blumgart duct-to-mucosa approach has been adopted. These changes introduce inherent bias and may have influenced the study's outcomes. The subjective nature of the assessment of pancreatic texture may also be another source of bias.

## Conclusions

This study showed that the Type D (according to the ISGPS system) or the two-risk factor group (according to the DPCG system) has the highest rate of postoperative complications after PD. On further analysis of the effectiveness of classification of pancreas-specific risk factors, including pancreatic texture and MPD diameter according to these classification systems, predictive accuracy was similar for CR-POPF; however, the DPCG classification with the simpler three-tier system is easier to apply in practice.
